# Nanoscale resolved mapping of the dipole emission of hBN color centers with a scattering-type scanning near-field optical microscope

**DOI:** 10.1515/nanoph-2024-0554

**Published:** 2025-02-07

**Authors:** Iris Niehues, Daniel Wigger, Korbinian Kaltenecker, Annika Klein-Hitpass, Philippe Roelli, Aleksandra K. Dąbrowska, Katarzyna Ludwiczak, Piotr Tatarczak, Janne O. Becker, Robert Schmidt, Martin Schnell, Johannes Binder, Andrzej Wysmołek, Rainer Hillenbrand

**Affiliations:** Institute of Physics, 9185 University of Münster, Wilhelm-Klemm-Str. 10, 48149 Münster, Germany; Department of Physics, University of Münster, Wilhelm-Klemm-Str. 9, 48149 Münster, Germany; Chair in Hybrid Nanosystems, Nano-Institute Munich, Department of Physics, Ludwig-Maximilians-Universität München, Königinstr. 10, 80539 Munich, Germany; Attocube Systems AG, Eglfinger Weg 2, 85540 Haar, Germany; CIC nanoGUNE BRTA, Tolosa Hiribidea 76, 20018 Donostia-San Sebastian, Spain; Faculty of Physics, University of Warsaw, ul. Pasteura 5, 02-093 Warsaw, Poland; IKERBASQUE, Basque Foundation for Science, 48013 Bilbao, Spain; Department of Electricity and Electronics, UPV/EHU, 48940 Leioa, Spain

**Keywords:** near-field spectroscopy, photoluminescence, hexagonal boron nitride, color center

## Abstract

Color centers in hexagonal boron nitride (hBN) are promising candidates as quantum light sources for future technologies. In this work, we utilize a scattering-type near-field optical microscope (s-SNOM) to study the photoluminescence (PL) emission characteristics of such quantum emitters in metalorganic vapor phase epitaxy grown hBN. On the one hand, we demonstrate direct near-field optical excitation and emission through interaction with the nanofocus of the tip resulting in a subdiffraction limited tip-enhanced PL hotspot. On the other hand, we show that indirect excitation and emission via scattering from the tip significantly increases the recorded PL intensity. This demonstrates that the tip-assisted PL (TAPL) process efficiently guides the generated light to the detector. We apply the TAPL method to map the in-plane dipole orientations of the hBN color centers on the nanoscale. This work promotes the widely available s-SNOM approach to applications in the quantum domain including characterization and optical control.

## 1 Introduction

Color centers in hexagonal boron nitride (hBN) have emerged as important quantum light sources due to their stable and bright single-photon emission at room temperature [1], [2], [3], [4] as well as their compatibility with photonic and electronic technologies [5], [6], [7], [8], [9]. Due to these properties, they are promising candidates for applications in quantum communication, quantum computation, and sensing technologies, making the understanding and manipulation of their properties crucial.

Scattering-type near-field optical microscopy (s-SNOM) is an advanced imaging technique that surpasses the diffraction limit, facilitating optical measurements down to the nanoscale [10], [11]. Utilizing sharp metallic tips of an atomic force microscope (AFM), s-SNOM not only provides topographical data but also yields optical contrasts of local material properties [12]. Techniques such as tip-enhanced Raman spectroscopy (TERS) [13], [14], [15], [16], [17] and photoluminescence (TEPL) spectroscopy [18], [19], [20], [21], [22] complement s-SNOM [23], [24], [25] and are ideal for examining the unique photophysical properties of single-photon emitters (SPEs). For example, TEPL has been used to study the emission properties of individual hBN emitters [26], [27]. Specially designed tips with resonant plasmonic particles have also been applied to study single molecules [28], [29], [30], [31] and quantum dots [32], [33].

## 2 Results

In this study, we examine the dipole emission characteristics of color centers embedded in 30 nm thick hBN layers grown by metalorganic vapor phase epitaxy [34], [35], [36], [37] (MOVPE; see supplementary material (SM) Sec. S1 for details). The layers are grown on sapphire and transferred onto a gold substrate [38]. Our investigation utilizes a scattering-type near-field optical microscope (*neaSCOPE* from *n* *easpec/attocube*) employing a standard metallized AFM tip (*Arrow-NCPt* sourced from *NanoWorld*) illuminated by monochromatic laser light. The tip acts as an optical antenna, transforming the incident p-polarized [39] light into a highly focused near field at the tip apex, the so-called nanofocus. The nanofocus interacts with the sample leading to modified scattering from the tip and encoding local sample properties. In conventional s-SNOM operation, the elastically scattered light is recorded as function of sample position (note that the sample is scanned), yielding near-field optical images with a spatial resolution down to 10 nm [10], [11], [23], [25]. To supress background scattering, the AFM is operated in tapping mode and the detector signal is demodulated at a higher harmonic of the tip's oscillation frequency [12]. Here, we use the s-SNOM instrument to study PL from individual hBN color centers. To that end, the inelastically tip-scattered light is recorded with a grating spectrometer coupled to a CCD camera. Note that signal demodulation has not been possible with the use of a CCD camera so far. It may be achieved employing a photomultiplier tube or similar [40]. Importantly, our s-SNOM setup includes a high-quality, silver-protected off-axis parabolic mirror with a numerical aperture (NA) of 0.72, which optimizes the focusing and collection efficiency of the optical system and is crucial for the performed PL measurements.

### 2.1 Characterization of photoluminescence mapping

In our specific experiments, we employ the near-field optical microscope in tapping mode, with low oscillation amplitudes between 20 nm and 30 nm, to detect PL signals influenced by the presence of the metallic AFM tip (a detailed description of the measurement procedures is given in the SM Sec. S2). Note that we use standard metallic AFM tips (*Arrow-NCPt* sourced from *NanoWorld*). Throughout this study, we use a 532 nm (2.33 eV) laser for the optical excitation of the hBN color centers (examples with other wavelengths are given in the SM Sec. S3, Fig. S1).

**Figure 1:**
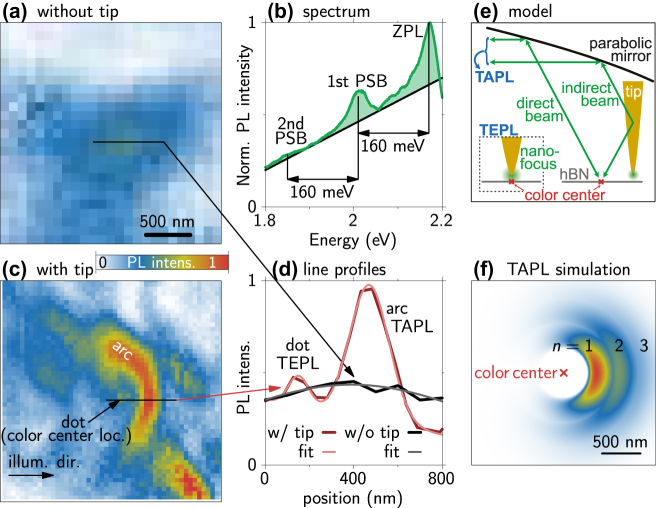
PL measurement of a single color center taken with an AFM tip. The images are shown with the same color bar for better comparison of the observed PL intensities. (a) PL intensity map without the tip showing a diffraction limited emission spot. (b) PL spectrum of the studied emitter recorded with an extended integration time inside the arc in (c). The zero-phonon line (ZPL) and optical phonon sidebands (PSBs) of 160 meV are marked as well as the broad background PL (black line). (c) PL map of the same emitter with the AFM tip showing two subdiffraction limit features marked as “dot” and “arc.” (d) Lineprofiles along the dashed lines in (a) in black and (c) in red (dark measurement, bright Gaussian fits). The fitted full widths at half maximum (FWHM) are 110 nm (dot), 209 nm (arc), and 1,418 nm (w/o tip). (e) Schematic of the interference between direct and indirect excitation/emission of the color center via the AFM tip (TAPL). Inset shows the nanofocus interaction at the location of the color center explaining the dot (TEPL). (f) Analytical reproduction of the TAPL arc in (c) applying the model in (e).

Figure 1a presents a typical far-field PL image of a single hBN color center recorded in the near-field optical microscope without a tip. At each pixel a PL spectrum is recorded and we integrate the intensity of the green shaded area in Figure 1b. Note that we determine this area under the peaks in practice by fitting as descriped in Ref. [4]. This procedure is performed for all PL maps shown in this work. The black line in Figure 1b indicates the broad PL background homogeneously observed over the whole MOVPE grown sample [41]. Figure 1a displays an elliptical maximum in the PL map with extensions of roughly 1 µm × 4 µm. This gives a benchmark for the optical resolution of the setup without AFM tip. To confirm that the light emission stems from a single color center, the corresponding PL spectrum is depicted in Figure 1b, which shows the typical asymmetric zero-phonon line (ZPL) and phonon sideband (PSB) characteristics [4], [42]. Note that we did not analyze the statistical properties of the light emission, since for our present study, it is not of relevance whether single-photon or classical emission is measured.

**Figure 2:**
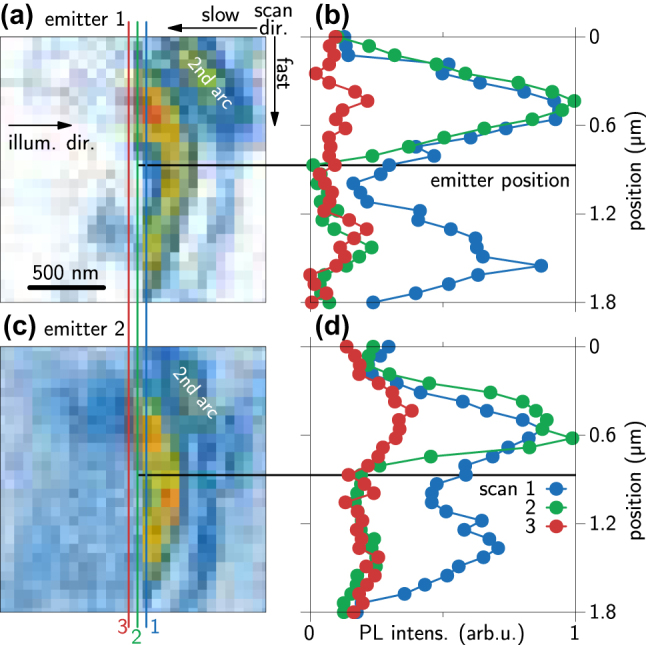
Near-field scan of two different emitters (a, c), with line profiles along the marked positions in (b, d). The measurement starts in the top right corner and line scan run vertically ending in the bottom left corner. Along the green scan line, the PL bleaches at the tip location of the emitter (dashed vertical line).

Notably, the PL map of the color center from Figure 1a experiences a dramatic transformation when imaged with a metallic AFM tip, as shown in Figure 1c (see also the direct overlay of the two images in SM Fig. S2). Here, the laser is focused on the tip generating the near-field nanofocus on the size of the tip apex (typically around 30 nm). Consequently, the sample is simultaneously excited via the far-field focus and the localized near-field focus. The tip is scanned over the sample and PL spectra are recorded at each position (pixel). The PL map in Figure 1c reveals two distinct features: (I) a circular symmetric emission hotspot, which we will refer to as “dot,” and (II) a more pronounced “arc” around the dot. It is important to note that this specific dot+arc pattern is consistently observed across many imaged hBN color centers (see detailed discussion in SM Sec. S3). We will discuss the origins of the two features (I: dot and II: arc) in the following paragraphs.

(I) Origin of the dot structure: To quantify the spatial resolution when recording PL maps with the AFM tip, Figure 1d shows a line profile along the dashed line in (c) (dark red) and a fit with two Gaussians on a tilted background (bright red). From this, we extract a full width at half maximum (FWHM) of 110 nm for the dot, which is clearly below the diffraction limit for far-field experiments. Therefore, we identify the dot as a result of the direct near-field interaction between the nanofocus at the tip apex and the color center leading to TEPL. The inset of Figure 1e schematically illustrates this explanation. For reference, the black line in Figure 1d shows a line profile along the dashed line in Figure 1a (without AFM tip) and the Gaussian fit reveals a FWHM of around 1,400 nm being the resolution in the setup without tip.

(II) Origin of the arc structure: The prominent arc structure has a diameter of around 1,000 nm and a FWHM cross section of 209 nm (extracted from the line profile and fit in Figure 1d). We can explain this feature by constructive interference between direct beams to/from the color center and those scattered from the AFM tip (indirect beam) as sketched in Figure 1e. To distinguish this effect from the near-field interaction leading to TEPL (dot), we call it tip-assisted photoluminescence (TAPL). To verify our explanation, we use a simple model to calculate the interference depending on the tip location. Besides the interference condition, the model includes an incidence/collection angle and interference widths, described by Gaussian intensity distributions, around this angle. We further take the NA of the parabolic mirror for the focusing angle and the movement of the color center with respect to the broadened focal point into account. A detailed description of the analytic interference model is given in SM Sec. S5, and the result for the TAPL signal, i.e., the arc, is shown in Figure 1f. We find an overall good agreement with the measurement and the higher interference orders leading to the arc replicas (*n* = 2, 3) show up in measurements with high optical powers (see Figure 2). This handy description gives an alternative explanation to the formation of standing surface waves between tip and emitter, suggested in Ref. [43]. In our model, the effect does not depend on the metallic surface of the substrate. Indeed, we find similar PL maps of emitters in hBN transferred onto Si/SiO_2_ substrates (see SM Fig. S1c). Note, that the direct nanofocus interaction with the color center resulting in the dot, i.e., TEPL, is not included in the model.

To finish this initial characterization of our PL maps, we come back to the experiment in Figure 1c and have a closer look at the intensities of the TEPL and TAPL features, dot and arc, respectively. Here and in all measurements, we find that the TEPL intensity of the dot is significantly weaker than the TAPL intensity of the arc. Thinking of the term “enhanced” in TEPL, this low intensity in the dot might appear counterintuitive. On the one hand, this could be caused through quenching effects by the proximity of the metallic AFM tip [27], [29]. On the other hand, the weak intensity of the dot could stem from averaging of the PL signal during the vertical tip movement in tapping mode. The tip oscillates with an amplitude of 20 nm, and we average the PL signal for 0.5 s. Therefore, the TEPL signal only contributes when the tip is in close proximity to the color center and rapidly diminishes for higher tip positions owing to the exponential distance dependence of the tip’s near field. This results in a reduced time-averaged TEPL signal. Conversely, the interference between the direct and indirect beams in TAPL (arc) depends only weakly on the tip height, thus contributing strongly to the overall PL signal during the whole tip oscillation cycle, resulting in the rather strong TAPL signal of the arc.

Finally, we compare the recorded PL intensities with and without the AFM tip. By spatially summing over the collected light intensity in the PL map in Figure 1a without tip and the PL map in Figure 1c with the tip (see SM Sec. S4 for details), we can estimate that we achieve an overall sixfold increased efficiency in this example. Note that the peak intensities differ by a factor of two (see Figure 1d). This demonstrates that the interference effect in TAPL due to the presence of the AFM tip contributes to the enhancement via two effects: (A) The optical excitation of the color center becomes more efficient and (B) the PL from the color center is guided efficiently into the collection angle of the parabolic mirror. Consequently, through this TAPL mapping, we can identify sample locations where metal antennas can be positioned to help guiding light toward an absorption center and guiding emitted light efficiently to a detector.

### 2.2 Demonstration of TEPL: bleaching of color centers

It is known since the first studies on hBN color centers, that optical excitation with too high intensities results in bleaching of the emission [44]. In Figure 2, we use this fact to demonstrate efficient optical driving through the nanofocus at the tip apex, i.e., TEPL. We show PL maps taken with an AFM tip of two emitters (a, c) with an increased optical excitation power of 500 µW (150 µW is used for the other measurements) and selected line profiles along the scan direction of the AFM in (b, d) marked by the colored lines in (a, c). The spectra are acquired in vertical lines starting from the top right to the bottom left. For both color centers, the emission bleaches almost entirely at exactly the position where we expect their locations (dashed black line, position of the dot). The line scan before reaching the emitter (blue) covers the double peak structure of the arc and the following scan after bleaching the emitter (red) is nearly entirely flat. The green scan line across the emitter location marked by the left end of the black dashed line still covers the first maximum of the arc but does not show the second one. This indicates that the emission gets bleached when the near field of the tip directly interacts with the color center. From the dot structure in the PL map of Figure 1c, we can conclude that the emitter interacts with the nanofocus of the tip and that the enhanced field at the tip apex leads to TEPL. By increasing the laser power, both the far- and near-field illumination of the emitter increase, eventually reaching its bleaching threshold. Owing to the field enhancement at the tip apex, the threshold is first surpassed when the emitter comes into the near-field nanofocus below the tip apex. Since bleaching is an irreversible process, unlike quenching, TAPL is observed only before (but not after) the emitter is exposed to the nanofocus. Note, that in the TAPL situation, i.e., in absence of tip-emitter near-field interaction, the local intensity acting on the emitter is still below the bleaching threshold of the emitter and PL is detected.

### 2.3 Utilization of TAPL: in-plane dipole emission mapping

**Figure 3:**
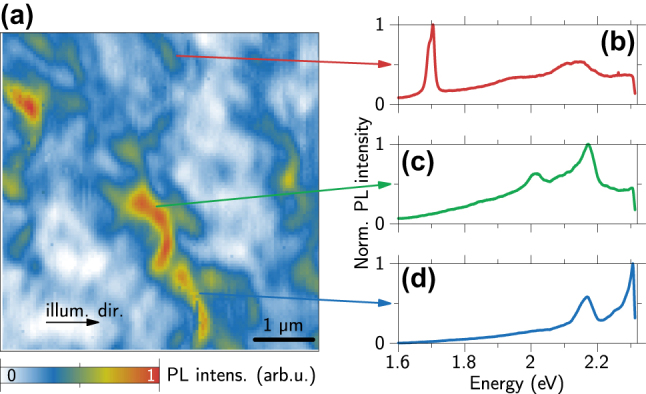
PL scan of serveral color centers in a 6 µm × 6 µm area. (a) Larger PL map with several emitters of different intensities and slightly different arcs, the black arrow marks the illumination direction. (b–d) PL spectra of color centers at the marked positions in the sample with long integration times of 5 s to improve the signal-to-noise-ratio. The spectrum in (c) is the same as in Figure 1b.

Figure 3 displays a PL overview map taken with an AFM tip spanning 6 µm × 6 µm with a pixel size of 50 nm. This map captures several emission centers, each distinguished by the characteristic arc. The same map with marked color center locations according to the arcs is given in SM Fig. S5. Note that the dot structure associated with TEPL is not visible for every color center. One reason could be that the emitter is located deep in the sample and cannot be reached with the near-field nanofocus. The PL spectra of three exemplary color centers recorded with long 5 s integration times are plotted in Figure 3b and c. The most prominently visible color center is located in the middle, which is the same as studied in Figure 1. A corresponding AFM height profile is available in SM Fig. S6. From this measurement, we cannot identify a correlation between the morphology of the sample and the appearance of color centers. Note, that from the variation in TAPL intensities, we cannot conclude different overall brightnesses of the color centers. As we will show in the following, the main reason is their variation in dipole orientation with respect to the illumination direction.

In the following, we demonstrate how the TAPL maximum can be utilized to determine the in-plane dipole orientation of a single color center in hBN. By systematically rotating the sample relative to the illumination direction, we monitor changes in the same emitters’ brightness. The respective AFM images from which we determine each rotation angle are shown in SM Fig. S7. The color centers are identified in each measurement by their location in the AFM and PL images and their spectral shape/ZPL energy. For each sample rotation, we record a PL map with the AFM tip and analyzed the position and spectra of the arc from four different color centers. For better comparability, we use the same tip for the entire measurement series.

**Figure 4:**
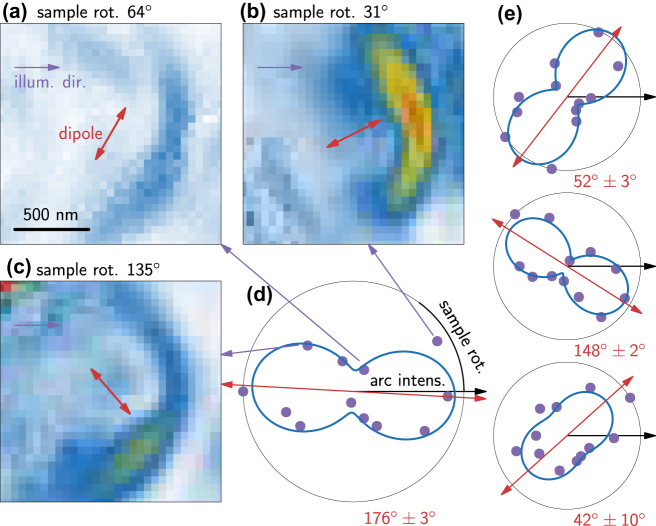
Mapping of the dipole orientation by sample rotation. (a–c) PL intensity maps (integrated peak area from fit) for different angles between illumination direction (violet arrows) and sample orientation (red arrows). (d) Polar plot of maximum PL intensities in the arc as a function of sample rotation (violet dots) with dipole fit (blue curve, see SM Sec. S8). (e) Dipole emission patterns of three other color centers in the same sample.

In Figure 4a–c, we show three example PL maps of the emitter from Figure 1c with different sample orientations, i.e., different illumination directions. The sample rotation for each map is stated above, and the red arrows indicate the dipole orientation (determined in Figure 4d) in relation to the illumination (violet arrows).

We find that the overall integrated emitter’s TAPL intensity of the arc strongly depends on the illumination direction (violet arrows), where (b) shows a large, (c) a medium, and (a) a small intensity. Figure 4d displays the extracted peak intensities inside the arc as a function of illumination direction in a polar plot, where the violet dots (experimental data) are fitted with a dipole pattern (blue). The plot reveals a clear dipolar emission characteristic for the color center with an orientation of 176° ± 3°. The other studied color centers in Figure 4e show different in-plane dipole orientations with 52° ± 3°, 148° ± 2°, and 42° ± 10°. We added these dipole orientations to SM Fig. S5. The nonvanishing intensity at orthogonal orientation between illumination direction and color center dipole can be explained by the emitter’s out-of-plane dipole component known from literature [45], [46], [47]. These results already demonstrate that this method provides a reliable way to map hBN color centers on the nanoscale and simultaneously gain insight in their optical properties. However, to find quantitative correlations between lattice orientation and emitter dipole, a larger set of emitters needs to be investigated in single crystal samples, which goes beyond the scope of this work. While we clearly recognize the arc in all three examples in Figure 4a–c, also the intensity distribution within the arc varies with the illumination angle. This could be related to the dipole orientation with respect to the crystal lattice, but again needs a dedicated systematic study on single crystal samples. In addition, the TAPL signal could be altered by details of the tip geometry (see Figure 1e and f). From the perspective of positioning nanoantennas near emitters to improve their excitation and PL (see end of Section 2.1), our TAPL dipole mapping results demonstrate that the antenna needs to be positioned aligned with the in-plane dipole orientation of the source for maximum efficiency.

## 3 Conclusions

In this study, we have demonstrated the utility of a scattering-type near-field optical microscope (s-SNOM), operated in tapping mode, for measuring the photoluminescence (PL) emission of color centers in hBN. The presence of the standard metallic AFM tip has two effects on the PL maps of the light emission centers. (I) The direct interaction between the near-field nanofocus at the tip apex and the color center leads to a subdiffraction limited emission spot and tip-enhanced PL (TEPL) at the location of the emitter. In the future, demodulation of the PL signal could be employed to isolate the TEPL signal from the PL background and improve the spatial resolution [48], [49]. However, with the current setup using a CCD camera this ist not possible., this limitation could be overcome by using a single-photon counter/photomultiplier tube. (II) The far-field interference between direct excitation/PL emission of the color center and the beam that is scattered from the metal tip results in a significant increase in detected PL intensity, which we call tip-assisted PL (TAPL). This effect manifests in an arc PL intensity around the color center and demonstrates that the emission direction from the color center can be controlled by the presence of the metal tip. Perspectively, this finding can be used to guide single photons generated by nanostructures via a metallic antenna. In addition, we have used the TAPL signal to map the dipole orientation of hBN color centers.

We note that the commercial s-SNOM setup used in this work is not uniquely optimized for TEPL measurements [27], [28], [30] but was originally designed for IR s-SNOM and nano-FTIR measurements [10], [12], [50], [51]. Our implementation of TEPL and TAPL (possible due to the high NA parabolic mirror), and the demonstration of novel functionality in the form of dipole mapping, renders valuable progress in the development of a s-SNOM operating with multispectral and multimessenger nanoprobes [52].

While we used polycrystalline hBN grown by metalorganic vapor-phase epitaxy (MOVPE), which supports a high density of color centers, our technique can also be applied to exfoliated hBN. This would allow to correlate the dipolar emission direction with the crystallographic axes of the material, potentially addressing the question of whether the SPEs originate from defects [53], [54] or are molecule-related [55]. In the latter case, no correlation between the lattice orientation and the emission direction would be expected, while real lattice defects should have a strong correlation.

Our introduced methodology opens new avenues for understanding the underlying physics of color centers in hBN and enhances the capabilities of nanoscale optical microscopy. Perspective applications obviously include subdiffraction limit control of other quantum excitations in semiconductors, e.g., excitons.

## Supplementary Material

Supplementary Material Details
