# Supplementary material for: Nanoscale resolved mapping of the dipole emission of hBN color centers with a scattering-type scanning near-field optical microscope
Source: Nanophotonics. 2025 Feb 7;14(3):335–42. doi: 10.1515/nanoph-2024-0554 (PMC11831392; doi:10.1515/nanoph-2024-0554)
Supplement: Supplementary file 1 — Supplementary Material Details [file j_nanoph-2024-0554_suppl_001.pdf]

I. Niehues et al.

Nanoscale resolved mapping of the dipole emission of hBN color centers with a scattering-type scanning near-field optical microscope (*Supplementary Material*)

S1 MOVPE grown hBN

Boron nitride was grown by Metalorganic Vapor Phase Epitaxy (MOVPE) on 2-inch sapphire c-plane substrates with a 0.6° off-cut angle [1], using an *Aixtron CCS* 3×2" system. Triethylboron and ammonia served as the boron and nitrogen precursors. A two-stage epitaxy approach [2] was employed, comprising a 10-minute Continuous Flow Growth (CFG) buffer layer and a subsequent 60-minute Flow-rate Modulation Epitaxy (FME) layer growth. The initial temperature of 1300°C (measured by an *ARGUS* optical pyrometer) was continuously increased until it reached 1400°C within 2 minutes after the start of the FME stage and was maintained at this level until the end of the process.

The samples were transferred onto other substrates by using a water-based transfer method [3]. To this end, the samples were immersed into a deionized water / isopropanol solution which releases the whole layer from the sapphire substrate. The solution can penetrate the interface between the hBN layer and the sapphire substrate, because the high-temperature growth of hBN on sapphire yields a characteristic mesh of wrinkles originating from different coefficients of thermal expansion [4, 5]. The floating hBN layer can be transferred on other substrates by immersing the target substrate into the liquid below the floating layer and gently lifting the substrate out of the solution. The transfer process allows the hBN layer to relax and the wrinkle pattern disappears. As target substrates, commercial Si/SiO_2 substrates (90 nm SiO_2 thickness) or gold coated sapphire substrates were used. The gold layer was deposited using sputtering techniques with layer thicknesses between 45-100 nm.

S2 Scattering-type near-field optical microscope setup

Our study employs a custom-designed scattering-type scanning near-field optical microscope (*Neascope* from *Neaspec/Attocube*). This system is operated in tapping mode AFM using standard platinum-iridium tips that feature a 30 nm tip apex diameter (*Arrow-NCPt* sourced from *NanoWorld*), ensuring precise interaction with the sample surface. The s-SNOM setup includes a high-quality, silver-protected off-axis parabolic mirror with a numerical aperture (NA) of 0.72, which optimizes the focusing and collection efficiency of the optical system. The microscope is equipped with a 532 nm laser (*Cobolt 04 series*, *Hübner*), which provides the necessary excitation for photoluminescence and Raman spectroscopy. In addition, a 561 nm laser and a 633 nm laser (*Cobolt 08 series*, *Hübner*) are used. Integrated into the setup is an *Andor Kymera* spectrometer with an *iDus* camera, which enables detailed spectral analysis across a wide range of wavelengths.

S3 PL measurements

The sample is initially brought into contact with the tip. The p-polarized 532 nm laser is first focused on the sample alone (without the tip involved) to maximize the Raman/PL signal, ensuring that the laser is precisely focused on the sample surface. Following this, the parabolic mirror is adjusted in the xy-plane to direct the laser onto the AFM tip without altering the z-axis positioning.

Proper alignment of the laser on the AFM tip is crucial, and this is achieved by maximizing the near-field signal (elastically scattered laser light) in the 3rd and 4th demodulation orders. It is worth noting that the near-field amplitude can be further increased by focusing the laser higher up on the tip (by moving the parabolic mirror in the $+z$-direction) than on the sample. However, it has been found that this is not the optimal position for our PL measurements. Once the laser is correctly aligned on the AFM tip, PL spectra are recorded using the typical measurement parameters:

- AFM tapping amplitude between 20 nm and 30 nm
- Laser power: 150 µW at the tip
- Integration time of 0.5 seconds.

It is important that the obtained spectra include both near-field and far-field contributions and are not demodulated.

In Figures S1(a, b) PL maps with different excitation laser wavelengths are shown and (c) shows a measurement on a Si/SiO_2 substrate. The measurements in the main text are performed with a 532 nm excitation wavelength and on a Au substrate.

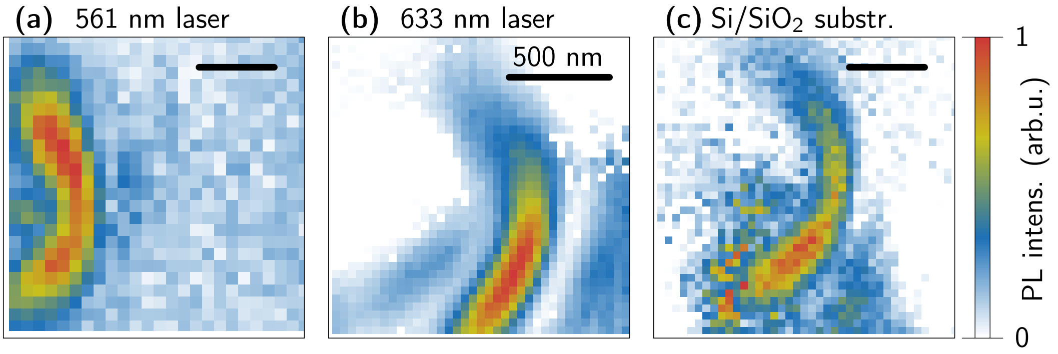

Fig. S1: PL maps taken with the AFM tip under varying conditions. (a, b) Excitation laser wavelength 561 nm and 633 nm, respectively, on a gold substrate (same sample as in the main text). (c) 532 nm excitation on a silicon substrate (hBN from the same growth process as the other sample but transferred to Si/SiO_2).

Figure S2 shows an overlay comparison between the PL image taken without the AFM tip (blue, same as Figure 1(a) in the main text) and the same image taken with the AFM tip (red, same as Figure 1(c) in the main text). This again confirms the improved resolution of the spatial PL scan and the more precise localization of single color centers. The contour lines in Figure S2(b) highlight the localization of the color center in the near-field image at the isolated black solid area compared with the blue outlined area from the image without tip. Note that we used a value of 0.7 for the blue contour of the far-field image without the tip. This value can directly be referred to the overall maximum PL intensity. We used a lower value of 0.43 to mark the dot in the near-field image because the overall maximum PL value lies in the arc and the dot maximum is smaller.

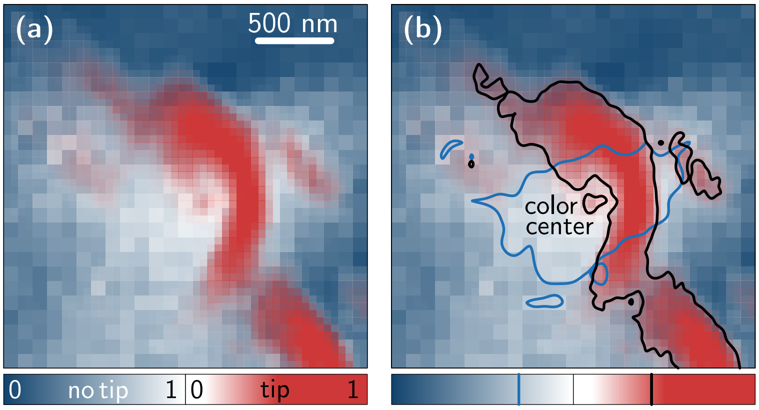

Fig. S2: Comparison between Figures 1(a) and 1(c) from the main text. (a) PL image taken without AFM tip in blue overlayed with the same image taken with AFM tip in red. (b) Same as (a) but with added contour lines. The blue contour line of the far-field image is plotted at 0.7 of the global PL maximum, while the black line of the near-field image is plotted at 0.43. Note that we used a lower value to mark the dot in the near-field image due to the higher PL values in the arc. The position of the color center is marked by the isolated black solid area in the middle of the image.

S4 Quantification of the PL enhancement

In Figure S3 we show how the spatial areas used for the spatial intensity summation are selected. (a, c) show the same results as Figures 1(a, c) in the main text and the dashed circles mark the areas used for the intensity summation. (b, d) show the actual data used for summation. We need to select the same sample areas for this spatial integration because we find regions with increased count rates stemming from different emitters in the near-field image in Figure S3(c), which we need to neglect. Finally, after summing over all pixels in (b) and (d) we find count numbers of 1043 and 6337 for PL without tip and PL with tip, respectively. This means an overall increase in detection efficiency of more than six times.

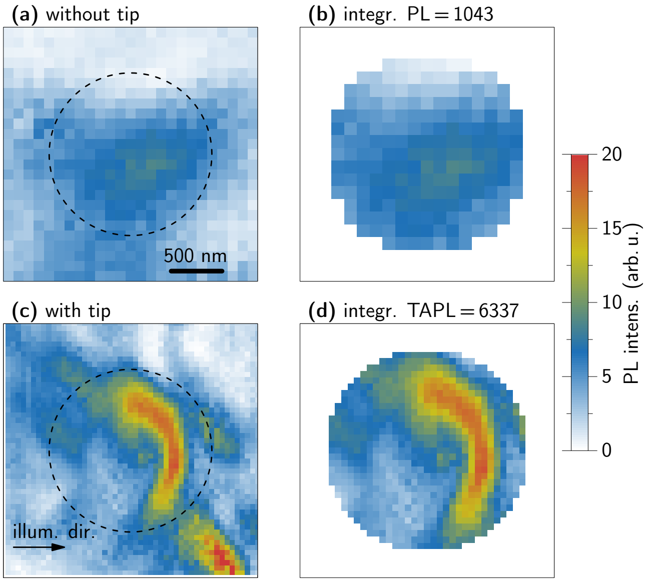

Fig. S3: PL intensity integration. (a, c) are the same measurements as Figures 1(a, c) in the main text. (b, d) show the respective circular cuts (dashed lines in (a, c)) for spatial intensity summation.

S5 Simulation of tip-assisted light emission

If we consider the situation in Figure S4(a) two beams are emitted from the color center: One directly hitting the collecting parabolic mirror, the other being scattered from the tip (experiencing a phase jump φ) and then running parallel to the first one into the mirror. These two beams will interfere constructively under the condition:

$$d\left(\alpha\right) = \frac{n - \sin\left(\varphi\right)}{2\sin\left(\alpha\right)}\lambda\,, \qquad n \in \mathbb{N}\,, \tag{S1}$$

where d is the distance between tip and emitter and λ is the emitted photoluminescence wavelength.

The efficiency of the scattering will depend on the orientation of the light polarization p_l with respect to the symmetry axis of the tip, i.e., the orientation of the scattering dipole p_s, such that the intensity of the detected light will be proportional to

$$I_\mathrm{scat} \sim \cos^2\left(\alpha\right)\ . \tag{S2}$$

Next, we take the interference angle σ and the focusing angle β into account. We model this by a Gaussian intensity profile around of width σ around β. This adds an angle-dependent intensity profile for the detection of

$$I_\mathrm{det}^{(z)} \sim \exp\left[-\frac{\left(\alpha-\beta\right)^2}{2\sigma^2}\right]\ . \tag{S3}$$

To get a full scan of the xy-plane we additionally need to add the focusing as sketched in the inset. This contributes an intensity scaling with

$$I_\mathrm{det}^{(xy)} \sim \exp\left(-\frac{\xi^2}{2\theta^2}\right)\ , \tag{S4}$$

which roughly corresponds to the NA of the parabolic mirror. This in-plane widening of the interference pattern additionally accounts for the non-vanishing radius of the tip. As the mirror and tip positions are fixed and the sample is moved in the scan, the focal point moves with respect to the emitter, such that we need to add an additional intensity scaling according to this movement according to

$$I_\mathrm{focus}^{(d)} \sim \exp\left(-\frac{d^2}{\lambda^2}\right)\ . \tag{S5}$$

For the overall intensity

$$I_\mathrm{PL} \sim I_\mathrm{scat} I_\mathrm{det}^{(z)} I_\mathrm{det}^{(xy)} I_\mathrm{focus}^{(d)} \tag{S6}$$

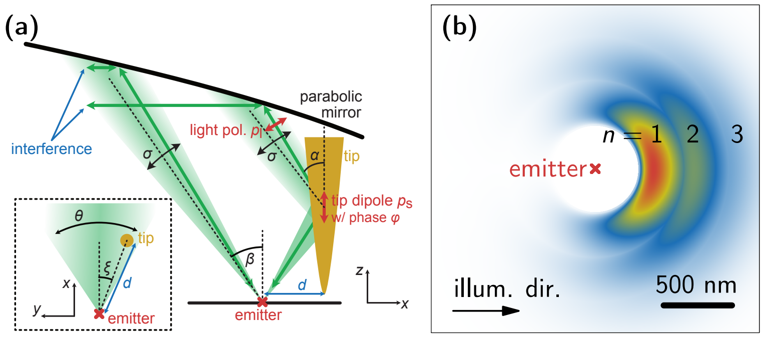

Fig. S4: (a) Schematic picture of the interference between direct emission from the emitter and indirect emission via scattering from the tip dipole. (b) Simulation of tip-assisted light emission with $\lambda = 650$ nm, $\varphi = \pi$, $\sigma = 0.1\pi$, $\beta = 0.25\pi$, $\theta = 0.3\pi$.

we get the result in Figure S4(b) and clearly see the arc for $n = 1,\ 2,\ 3$ with decreasing intensity. Note that we did not additionally include the direct emission from the color center, i.e., the central dot, in the image as it is no part of this model. The parameters are given in the caption. Arcs with higher n are sometimes seen experiment if the intensity and signal-to-noise-ratio are high enough (see Figure 2 in main text).

S6 Color center localization

Figure S5 shows the same PL map as in Figure 3 of the main text. We include red crosses where color centers are located according to the curvatures of the arcs in TAPL. Note that the dot from TEPL is not visible for all color centers. One reason could be that the emitter is located deep in the sample and cannot be reached with the nanofocus. In addition, we added the dipole orientations for the four color centers studied in Figure 4 of the main text as red arrows.

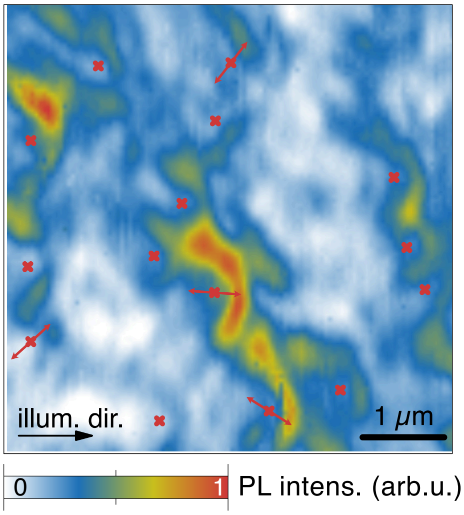

Fig. S5: Same PL map as in Figure 3(a) in the main text with color center locations (red crosses) estimated from the arcs. The double arrows show the dipole orientation of the color centers studied in Figure 4 in the main text.

S7 Atomic force microscopy image

Figure S6 shows the height profile of the samples studied in PL in Figure 3 of the main text. Both images were taken simultaneously. The morphology of the AFM images shows no strong inhomogeneities within the hBN flake. Therefore, from this measurement we cannot identify a correlation to the appearance of color centers and for example strain.

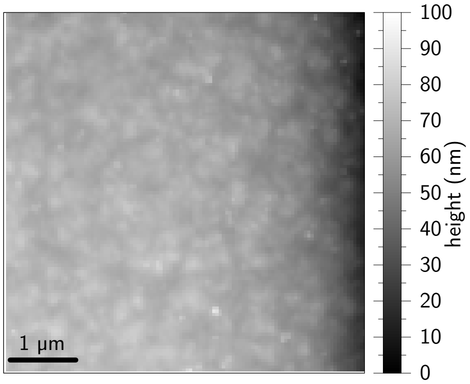

Fig. S6: Atomic force height profile of the sample corresponding to the PL map in Figure 3 in the main text.

S8 Determining the sample rotation angle

Figure S7 shows the height profile of the sample for all different rotation angles used in Figure 4 of the main text. The rotation angle between sample and illumination direction was determined by tracing the flake edge (red dashed line) and a spot (most likely dirt) on the sample surface (red triangle).

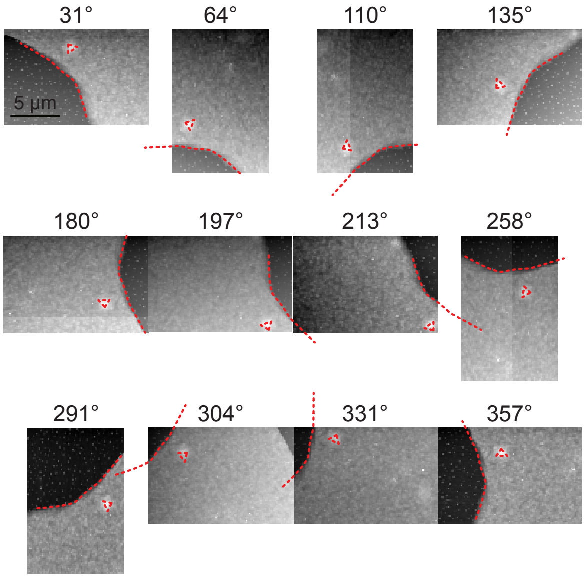

Fig. S7: Atomic force height profile of the sample for different sample orientations with respect to the illumination direction.

The angle dependence of the PL intensity in Figure 4 of the main text is fitted with a standard dipole emission patter of the form

$$I_{\text{dipole}} \sim \cos^2(\phi - \phi_0) + I_0 \ , \tag{S7}$$

where ϕ is the sample rotation, ϕ_0 is the orientation of the color center's dipole and I_0 is a constant PL intensity, potentially stemming form the out-off-plane dipole component of the color center (see main text). Note that our analytic interference model from Section S5 does not contain any contribution that impacts the in-plane polarization of the light making it compatible with this standard fit of the dipole pattern.

References

[1] M. Tokarczyk, A. K. Dąbrowska, G. Kowalski, R. Bożek, J. Iwański, J. Binder, R. Stępniewski, and A. Wysmołek, "Effective substrate for the growth of multilayer h-BN on sapphire—substrate off-cut, pre-growth, and post-growth conditions in metal-organic vapor phase epitaxy," *2D Mater.*, vol. 10, no. 2, p. 025010, 2023.

[2] A. K. Dąbrowska, M. Tokarczyk, G. Kowalski, J. Binder, R. Bożek, J. Borysiuk, R. Stępniewski, and A. Wysmołek, "Two stage epitaxial growth of wafer-size multilayer h-BN by metal-organic vapor phase epitaxy–a homoepitaxial approach," *2D Mater.*, vol. 8, p. 015017, 2020.

[3] K. Ludwiczak, A. K. Dąbrowska, J. Kucharek, J. Rogoża, M. Tokarczyk, R. Bożek, M. Gryglas-Borysiewicz, T. Taniguchi, K. Watanabe, J. Binder, W. Pacuski, and A. Wysmołek, "Large-area growth of high-optical-quality $MoSe_2$/hBN heterostructures with tunable charge carrier concentration," *ACS Appl. Mater. Interfaces*, vol. 16, no. 37, pp. 49 701–49 710, 2024.

[4] D. Chugh, J. Wong-Leung, L. Li, M. Lysevych, H. H. Tan, and C. Jagadish, "Flow modulation epitaxy of hexagonal boron nitride," *2D Mater.*, vol. 5, no. 4, p. 045018, 2018.

[5] J. Binder, A. K. Dabrowska, M. Tokarczyk, K. Ludwiczak, R. Bozek, G. Kowalski, R. Stepniewski, and A. Wysmolek, "Epitaxial hexagonal boron nitride for hydrogen generation by radiolysis of interfacial water," *Nano Lett.*, vol. 23, no. 4, pp. 1267–1272, 2023.
